# Wife beating refusal among women of reproductive age in urban and rural Ethiopia

**DOI:** 10.1186/s12914-017-0115-5

**Published:** 2017-03-16

**Authors:** Eshetu Gurmu, Senait Endale

**Affiliations:** 1 Center for Population Studies, College of Development Studies, Addis Ababa University, Addis Ababa, Ethiopia; 2 Population and Gender Research Unit, Institute of Development and Policy Research, Addis Ababa University, Addis Ababa, Ethiopia

**Keywords:** Wife beating, Gender based violence, Refusal, Women, Ethiopia

## Abstract

**Background:**

Wife beating is the most common and widespread form of intimate partner violence in Ethiopia. It results in countless severe health, socio-economic and psychological problems and has contributed to the violation of human rights including the liberty of women to enjoy conjugal life. The main purpose of this study is to assess the levels and patterns of wife beating refusal and its associated socio-cultural and demographic factors in rural and urban Ethiopia.

**Methods:**

The 2011 Ethiopian Demographic and Health Survey (EDHS) data based on 11,097 and 5287 women in the reproductive age group (i.e. 15–49 years) living in rural and urban areas, respectively,were used in this study. Cronbach’s alpha was used to assess the internal consistency of the measure of women’s attitudes towards wife beating. The Statistical Package for Social Sciences was applied to analyze the data. A binary logistic regression model was fitted to identify variables that significantly predict respondents’ refusal of wife beating. Separate analysis by a place of residence was undertaken as attitude towards wife beating vary between rural and urban areas.

**Results:**

The likelihood of refusing wife beating in Ethiopia was significantly higher among urban women (54.2%) than rural women (24.5%). Although there was a significant variations in attitude towards refusing wife beating among different regions in Ethiopia, increasing educational level, high access to media, age of respondents were associated with high level of refusal of wife beating. In contrast, rural residence, being in marital union, high number of living children, being followers of some religions (Muslim followers in urban and Protestants in rural) were associated with low level of refusal of wife beating.

**Conclusion:**

The findings of this study reveal that wife beating in Ethiopia is a function of demographic and socio-cultural factors among which age and educational attainment of respondents, number of living children, religious affiliation, marital commitment and region of residence play significant roles. As factors governing perceptions and behaviours of individuals and institutional settings appear to shape knowledge and attitude towards gender equity and equality, awareness creation and behavioural change initiatives should be considered to abolish violence against women.

## Background

Violence against women is ‘…any act of gender-based violence that results in, or is likely to result in, physical, sexual or mental harm or suffering to women… whether occurring in public or in private life’ [1]. The Beijing Platform for Action also defines any form of violence against women as the violation of the human rights that impedes the achievement of the objectives of equality, development and peace in any society [2]. Violence against women by an intimate partner is also identified as one of the most common and widespread forms of gender based violence (hence forth, GBV) in the world [3]. Global survey on violence against women conducted by World Health Organization revealed that 30% of women (at least one in three) who had been married to or otherwise partnered with had experienced either physical or sexual violence by intimate partners [4]. The prevalence is; however, the highest in Africa in which about 37% of women have been victims of either physical or sexual violence by an intimate partners [3, 4]. Ethiopia is one of the African states in which the highest rate of intimate partner violence prevails. In a 2005 WHO report, about 71% of Ethiopian women who had been married to or otherwise partnered with intimate had experienced either physical or sexual violence by their partners. Out of them, about 35% had experienced at least one form of severe physical mistreatment, *inter alia*, being hit with a fist or something else, hard kicked, forcefully dragged, beaten, flogged, chocked, burnt, threatened with weapons [5, 6].

Domestic violence has brought greater impacts on women’s autonomy and sense of worth. It has also impeded in their ability to care for themselves and for their children [5]. It has reduced their opportunities and rights for work, mobility and participation in education and training, in community activities and in the wider social network [7]. Domestic violence against women has caused major traumatic disabilities and heart smiting deaths among women of reproductive age in both developed and developing countries in which the enigma in developing countries is estimated to account for 5% for their healthy year’s life loss [8, 9]. It also has serious repercussions on children’s life which has resulted in poor performances in schools, increased probability of delinquency, leaving homes early, risks of abuse and joining street life, engagement in substance abuse, attempting to commit suicide and disturbing the family, etc. [7, 10, 11].

Domestic violence has incurred immense economic costs on developing nations for prices and expenditures to prevent detect and offer health, social and legal services to countless survivors of this particular passion [9, 12]. The violence has caused many women in being absent from work, and the time and medication required to treat physical and psycho-social damages has also reduced workers’ productivity, earned income and effective utilization of accumulated human and social capital [11].

Despite taking different legal and policy measures to address violence against women (VAW), domestic violence, specifically wife beating, is still the highest as an acceptable norm in Ethiopia [6, 13]. The scholars identified that through ratified international documents and adopted national instruments such as Ethiopian Women Policy [14], the Revised Criminal Law [15], the Revised Family Law [16], Strategic Plan for an Integrated and Multi-Sectoral Response to Violence against Women [17], etc. actions have been taken though non-remedial to the quandary. There are two major reasons for the highest prevalence rate of wife beating in Ethiopia. The first is the nationwide existence and acceptance of hostile gender biased attitudes such as admitting domestic violence, in general, and wife beating, in particular, as normal and private matters. The second is the swift implementation of existing national laws to address GBV in the country [17].

Studies conducted on GBV revealed that men and women’s attitudes towards wife beating are strongly correlated to their exposure with intimate partners’ violence [11, 18]. Abrahams and colleagues’ study showed that men who knew and believed that wife beating is an acceptable norm were two times more likely to be engaged in intimate partner violence than their counterparts. It is thus possible to argue that wife beating in Ethiopia is mainly a function of the socio-cultural settings in which the communities are living and ineffective legal and protective actions being taken to overcome the problem.

Although the societal acceptance of wife beating is believed to be widespread in Ethiopia, empirical evidences on the extent of refusal of wife-beating and its explanatory factors are non-existent. No study, to the knowledge of the authors, has ever been undertaken to investigate the determinants of wife beating refusal in urban and rural Ethiopia. No documents that show its prevalence, patterns and determinants or endeavours of a contribution towards the initiatives to be taken or scaled up to eliminate the problem exist. This study, therefore, focuses on assessing the levels and patterns of wife beating refusal and its associated socio-cultural and demographic factors in rural and urban Ethiopia.

## Theoretical considerations

The general consensus among researchers about intimate partner violence is that they viewed it as a manifestation of unequal power relationship between men and women [19]. Intimate partner violence is reinforced by gender norms and values that put women in subordinate position relative to men [20]. Even though there are many theories on the causes and consequences of intimate partner violence, only three theories, namely, resource based power theory, feminist theory and exchange theory are reviewed as they seem to easily explain the situation of intimate partners’ violence in developing countries like Ethiopia.

### Resource based power theory

The resource-power interrelationship theory states that power within a family is a function of the ownership of resources within which members have access and decision making autonomy [21]. The theory further states that a person who brings more resources to the relationship possesses more power, and the opposite is true [22]. Hence, violence within a family would thus arise in a struggle to sustain such power balance and relationship [23]. There are, however, different arguments whether there is a direct relationship between the increasing gender equality and violence or not. Levinson, for example, strongly argued that the more resource a husband brings to a relationship, the more power he inspires to have, but the less likely he will resort to violence. However, he argues that if the man’s superior power is threatened by his wife’s access to education or job related resources, he may resort to violence to re-establish his status [24]. Hence, in societies where men’s power is eroded due to women’s increased access to non-monetary resources such as educational attainment and/or career mobility, there would be an increase in domestic violence as the power relationship goes beyond control of resources in the questionings egalitarian status [21].

Whaley, who studied the paradoxical relationship between gender inequality and rape came up with a new argument that questions the direct and consistent relationship between increasing gender equality and violence [25]. According to Whaley, gender equality leads to violence between couples only for shorter period of time—till the issue (s) leading to conflict would be sorted out by the couples or intimate partners through time, while an inverse relationship between gender equality and violence will be maintained over time. For Whaley, gender equality not only improves the social climate but also reduces the extent of conflict and it diminishes the levels of domestic violence due to increased mutual understanding and support [25]. In the same way, research conducted in Chengdu, the capital of Sichuan province in southwest China, came out with the result that the more economic resources the wife controls, the less likely she is to become a victim of GBV [26]. According to this theory, an increase in women’s access to resources leads to the ratification of gender equity and equality along with the freedom to withstand any sort of intimate partner violence including wife beating. The theory, in general, contemplates the condition under which domestic violence of intimate partner is refuted as a result of socio-economic development that erodes the reflection of traditional norms and values.

Although the resource based power theory explains the conditions under which access to resources accelerate or diminish GBV, it is criticized for ignoring cultural variables that are based on gender ideologies in perpetuating male dominance and intimate partner’s violence [27]. In traditional societies like Ethiopia, wife beating is also manifested as a mechanism of showing supremacy together with taking the full control of resources owned at the household level [24].

### Feminist theory

Feminists argue that throughout history, patriarchy with its gender ideology has created a society which puts men in dominant and women in subordinate positions [28]. Rigid social norms and cultural practices are the major instruments of patriarchal societies that perpetuate and maintain the myths of male superiority [29]. These patriarchal norms justify the use of violence to protect men’s ability to control women [30]. Wife beating is, thus, the central theme of a patriarchal ideology or a myth that perpetuates male authority through the use of force. According to a study conducted among women living in Toronto in late 1980s, Smith found that there is a positive correlation between belief in patriarchy and wife beating as men who scored higher approval attitudes towards gender based violence were found more likely to beat their wives than others [31]. For feminists, wife beating is considered as a function of gender inequality which mainly has been associated with a culture of violating women’s right to subjugate their position [24].

The feminist theory, on the other hand, recognizes the improvements in the protection of women’s right in modern societies where women’s active participation in the socio-economic system is increasing as a result of the changes in their educational, occupational, and political status. The theory claimed that improvements in the status of women diminish the rate of physical and sexual violence against women [32]. Unlike this claim, in traditional societies where the status of women is low and wife beating is high, there is still a violation of women’s rights which perpetuated in time with an ideology of familial patriarchy. A study conducted in India, for instance, revealed that husbands who had lower-income, less-educated, and were engaged in relatively low-status jobs were more likely to have beaten their wives than were more advantaged husbands [33].

Even though the importance of feminist perspective was well recognized in explaining the tragedy behind wife beating, it was also subjected to criticism by many scholars such as Dutton & Corvo, Dutton & Nicholls, and Jasinski for lacking empirical evidences in supporting the major points of argument and in attending a sufficiently large number of intimate partners’ violence due to the sole influence of socio-cultural settings [22, 34, 35]. Far beyond this, feminist theory is criticized for its inability to account for violence caused by women in both heterosexual and lesbian relationships, and for throwing deaf ears to alternative approaches to understand the causes and consequences of intimate partner’s violence [36, 37].

### Exchange theory

The basic premise of exchange theory is based on behavioural changes that are driven by rewards or punishments [38]. In this theory’s context, domestic violence including wife beating is very high in societies where the benefit to perpetrators is immense, but is very low where the penalties is high [24, 39]. In traditional societies where domestic violence is very common, charges facing those committing intimate partner violence is low [39]. In explaining the issue further, Gelles stated, taking domestic violence as a private matter, lack of interest in social institutions and agencies to intervene and little attention paid to the matter have made the occurrences of intimate partner violence a perpetuating mystification [40]. Moreover, cultural acceptance of the violence as one of the mechanisms of managing household conflict and maintaining order in the family system has increased the potential “rewards” of intimate partner violence [41]. Lower perceived costs of intimate partner violence, in general, and wife beating in particular, has led the incidence to be taken as normal and a less likelihood to resist the act.

Williams who tested the proposition of exchange theory using data from two national survey studies in United States found out that, along with other determinants such as lower social control and greater gender inequality, men who approved of hitting their partners (because of the lower perceived costs associated with it) were more likely to perpetrate violence against their wives [42]. Arthur and Clark, on the other hand, who did similar study in societies that impose punishments by laws against domestic violence and mechanisms to its effective enforcement, found out a lower level of domestic violence for the mere reason that it is legally bounded [30]. Evidences from the aforementioned empirical studies show that attitude towards intimate partner violence including wife beating is a function of rewards and punishments practiced in a given society as well. Thus, to reduce wife beating, rewards must be reduced by ending the social adoration of violence, and costs must be increased by setting strong legal and social sanctions against wife beaters [40, 41].

## Empirical research findings

Empirical research findings also state that socio-economic and demographic factors have their own specific influences on wife beating and the refusal against such act. Following are review of selected socio-economic and demographic factors having strong association with acceptance and refusal of wife beating.

### Woman’s educational attainment

According to WHO, education is the strongest demographic predictor explaining wife-beating attitudes [5]. Women’s odds of accepting wife beating gets reduced by more than half with higher education (over 12 years), compared to women with secondary (6–12 years) or primary education [33]. This implies that women with high level of education were found to refuse wife beating more than those with low level of education. A study conducted in seven Asian countries based on national representative data collected between 1998 and 2001 also revealed that better educated women were more likely to disapprove wife beating than less educated once [43]. Similar studies conducted in Israel [44], Korea [45], Vietnam [46] and seventeen sub-Saharan African countries (including Ethiopia) [47] had also came up with consistent findings.

### Age and marital status of woman

Refusal of wife beating is expected to significantly associate with age. Theoretically persons in the older age group are more likely to refuse wife beating than those in younger ages, and empirical evidences obtained from studies undertaken in different parts of the world showed that age was negatively associated with acceptance of wife-beating [43, 44, 48–50] . Similarly, a comparative study from 17 countries in sub-Saharan Africa revealed that intimate violence against women (i.e. wife beating) was widely acceptable under certain circumstances among women at younger than older ages [47]. Refusal of wife beating by older women is partly attributed to the support of children who tend to challenge the act of men on their mothers [33]. Married women were also found to justify wife beating more than single women in Nigeria [50] and other seven sub-Saharan African countries including Ethiopia [29] as the patriarchal system imposes male’s supremacy over women.

### Place of residence

Studies conducted in 17 sub-Saharan African countries, including Ethiopia [47] Nigeria [50], Zimbabwe [48] and Egypt [51] clearly indicate that women living in rural areas were more likely to justify intimate partner violence than those living in urban areas. The variation in the level of accepting wife beating between rural and urban women is partly attributed to the effect of traditional norms and values that perpetuate in rural areas but nearly diminishing in urban settings due to the atonally of modernization elements [29].

### Religion

Religion tends to clutch abundant moral values affecting the power relation between husband and wife. Women who are more religious are believed to hold conservative and traditional belief that stimulates husbands’ to abuse their wives [50]. In a research among seven sub Saharan African countries, even though no consistent relationship was observed between attitude towards wife beating and religion in all countries, compared to Catholic women, Muslims in Mali and Benin and followers of other religions in Zimbabwe were more likely to justify wife-beating [29]. A study in Ghana also revealed that compared to Christian women, Muslims and Traditional believers were more likely to approve physical violence against wives [52]. Unlike this, among women and men in the United States, being Catholic rather than non religious has been associated with high refusal of wife beating [53].

### Access to media

Due to variation in level of awareness about human right and the means to preserve one’s sovereignty, access to media information was inversely related to justification of beating a wife for one reason or another [50]. A study conducted in 17 sub-Saharan African countries, for instance, revealed that respondents with low access to media information were more supportive of wife beating than their counterparts who have much access to media information [47]. In the same way, a study conducted in Nigeria also indicated that watching television reduces the likelihood of justifying wife beating [50].

### Wealth status

Partly due to the possibility of getting access to some resources even if the marriage is going to dissolve, women living in wealthier households have the tendency to refute wife-beating [54]. A study conducted among seven Asian countries, for instance, showed that women in the poorest economic quintile were more likely to justify wife beating than those living in the richest quintile [43]. Consistent results were also obtained in studies conducted among 17 sub-Saharan African countries using the demographic and health survey data [47]. These findings are consistent with Goode’s resource theory dealing with domestic violence against women [21].

Given the complex nature of socio-economic and cultural settings governing the perpetuation of GBV including wife beating in Ethiopia, the aforementioned theoretical frameworks shall be taken a functional in analyzing and interpreting refusals of wife beating in rural and urban Ethiopia. The aforementioned theoretical arguments are believed not only to guide the study but also to synthesize the conditions under which it is operating.

## Methods

Inclusive measurement of attitude towards wife beating requires considering as many indicators as possible [29]. The available indicators measure the attitude of women in reproductive age towards accepting wife beating for reasons associated with household management and conjugal relationship [55]. Besides, in traditional societies like Ethiopia, wife beating is considered either as symbol of reflecting the ‘love’ that a person has for his wife or as a means of showing the man’s supremacy over his wife [6].

In this paper, the study is based on the analysis of Ethiopian Demographic and Health Survey (EDHS) Data in 2011 which is believed by the researchers as a national representative sample of women in the reproductive age. The survey was conducted based on sampling frames derived from the Population and Housing Census conducted by theCentral Statistical Agency of Ethiopia in 2007. *Kebeles*- the smallest administrative unit in the nation were used as primary sampling units; while, households constituted secondary sampling units. A total of 16 515 women in the reproductive age group (i.e. 15–49 years) were interviewed. The response rate for the survey was 95%. The data were collected through face-to-face interviews [55]. In this study, the questions included in the 2011 EDHS to collect information on wife beating were used to construct an index of refusal to wife beating. For this, refusal of wife beating was measured as a composite index consisting of the following items: a husband is justified in hitting or beating his wife if she (a) burns the food (b) argues with him (c) goes out without telling him (d) neglects the children, and (e) refuses to have sexual intercourse with him. Each item has a response of ‘Yes’ and ‘No’, and a value of ‘1’ is given if the woman accepts but ‘0’ otherwise. The composite index runs between ‘0’ when the woman refuses the hitting or beating at all and ‘1’ if she accepts the hitting or beating for any of the reasons. A woman is considered as ‘refusing wife beating’ if she responded ‘No’ to all of the five questions, and ‘accepting wife beating’ otherwise. The Cronbach alpha value of the index of attitude towards wife beating was 0.72 for rural and 0.78 for urban areas. The alpha values are of acceptable standard as both of them are greater than the minimum required level of 0.7 [56].

The independent variables were region, respondents’ age group, educational level, religion, work status, marital status, number of living children born to a woman and access to media as well as household wealth status. The percentage distribution of women’s attitude towards wife beating by socio-economic characteristics of the respondents was given using tabular presentations. The Statistical Package for Social Sciences (SPSS version 20) was used to analyse the data. A binary logistic regression model was fitted to identify variables that significantly predict respondents’ refusal of wife beating in Ethiopia having controlled other confounding variables. Separate analysis by a place of residence was undertaken as attitude towards wife beating vary between rural and urban areas. The multivariate analysis model was fitted using the actual (i.e. non-weighted) data.

## Results

There were considerable differences between rural and urban areas in the percentage distribution of women who had been refusing wife beating for any of the reasons. In urban areas, as it was found out, more than half (54.1%) of the respondents refused wife beating for any of the reasons; while, only about a fourth (24.5%) of the rural women had to refuse wife beating for any of the reasons (see Table 1, Panels I and III). The level of refusing wife beating in rural areas also varied across regions (Table 1, Panel I). That is, about 30% of rural women in Benishangul Gumuz, Oromia and Gamblla had refused wife beating, but in Somali, SNNP and Afar regions, less than 20% of the rural women had refused wife beating. In rural areas of the remaining regions, the percentage of women with refusing attitudes was between 20 and 30%. Unlike this, more than half (>50%) of urban women residing in Addis Ababa, Dire Dawa, Oromia, Harari, Benishangul Gumuz and Southern Nations, Nationalities and Peoples (SNNP) regions refused wife beating. Only 23% of women living in urban Somali, 37.4% of urban Amhara and 42.9% of urban Gambella had refused wife beating (Table 1 Panel III).

**Table 1:** Percentage distribution of women of reproductive age by their attitude towards wife beating

Variables	Classification	Rural		Urban	
		Refuse wife beating	Accept wife beating	Refuse wife beating	Accept wife beating
		I	II	III	IV
Region	Afar	19.8	80.2	48.7	51.3
	Amhara	21.3	78.7	37.4	62.6
	Oromia	29.8	70.2	59.2	40.8
	Somalia	13.3	86.7	23.0	77.0
	Ben-Gumuz	32.6	67.4	53.8	46.2
	SNNP	18.1	81.9	51.0	49.0
	Gambella	29.2	70.8	42.9	57.1
	Harari	21.1	78.9	53.3	46.7
	Addis Ababa	–	–	76.1	23.9
	Dire Dawa	27.8	72.2	62.0	38.0
	Tigray	27.5	72.5	47.1	52.9
Level of education	No Education	19.4	80.6	78.3	21.7
	Primary	29.4	70.6	61.9	38.1
	Secondary	49.5	50.5	52.0	48.0
	Higher	79.9	20.1	35.1	64.9
Age	15–24	28.4	71.6	53.8	46.2
	25–34	22.2	77.8	55.3	44.7
	35–49	21.5	78.5	53.1	46.9
Access to media	Not at all	21.1	78.9	23.8	76.2
	Sometimes	24.0	76.0	50.5	49.5
	Frequently	31.4	68.6	60.1	39.9
work status	Not working	24.2	75.8	56.4	43.6
	Family business	26.0	74.0	44.9	55.1
	Employed	23.6	76.4	54.0	46.0
Marital Status	Never married	35.5	64.5	57.8	42.2
	Currently in Union	20.4	79.6	53.3	46.7
	Ever married	26.7	73.3	45.9	54.1
Religion	Orthodox	26.0	74.0	56.4	43.6
	Protestant	20.4	79.6	52.3	47.7
	Islam	26.2	73.8	48.6	51.4
	Others	17.7	82.3	41.7	58.3
Number of living children	None	32.6	67.4	57.0	43.0
	1–2	23.0	77.0	56.2	43.8
	3–4	19.5	80.5	48.2	51.8
	5 and above	20.3	79.7	42.1	54.2
Household wealth Status	Low	22.8	77.2	39.6	60.4
	Medium	23.1	76.9	50.7	49.3
	High	27.8	72.2	70.7	29.3
Total		24.5	75.5	54.2	45.8

Source: computed by authors from EDHS 2011 Data set

Results of the study revealed that refusing wife beating was differed by educational attainment. Even though a direct relationship between educational level and refusal of wife beating was observed, there existed variation between rural and urban areas. More resistance to wife beating by education level was observed in urban areas though the level is nearly the same among those attaining higher educational levels. Likewise, an inverse relationship between refusal of wife beating and number of living children is observed in urban and rural areas though the proportion of refusal was substantially lower among women residing in rural areas (Table 1, Panels I and III).

Women with no access to media in both rural and urban areas had less resistance to wife beating (21.1 and 23.8%, respectively) (Table 1, Panels I and III). Unlike this finding, there was a wider variation among those who have frequent access to media in urban (60.1%) and rural (31.4%) areas. The proportion with a refusing attitude towards wife beating was also higher among women in frequent access to media in urban (50.5%) than in rural areas (24.0%) (Table 1 Panels I and III). Despite variations in the level of refusal to wife beating in urban and rural settings, the age of respondents had nearly shown similar patterns in both rural and urban settings.

Rural women who have different work and wealth status had nearly similar level of refusal to wife beating (Table 1, Panel I). In urban areas, however, about 70.7% of women residing in rich households refused wife beating which was higher than the proportion among the poor (39.6%). Refusal to wife beating was found to be the highest among urban never married women (57.8%); whereas the proportion of urban women who refused wife beating was lower (45.9%) among those who married previously. Differences in the attitude towards wife beating were also observed among urban women due to work status; that is, 56.4% of non-working urban women refused wife beating vis-à-vis 44.9% of urban women working in family business. Similarly, more than half (56.4%) of urban women following Orthodox religion refused wife beating while the proportion who refused wife beating declined to 41.7% among urban women who have been following traditional and other religions. Closer to half (48.6%) of urban Muslim women were also observed to refuse wife beating (Table 1 Panel III).

The multivariate results have also shown significant variations of refusing wife beating by region. In comparison with women living in Tigray region, rural women in Somlia, Afar, Harari and Amhara regions were less likely to refuse wife beating. Their refusal attitude has decreased by 60, 34, 32 and 29%, respectively, and the effect is statistically significant (Table 2, Panel II). Rural women living only in Benishangul Gumuz region were 41% more likely to refuse wife beating (*p* < 0.01). However, rural women living in Oromia, Gambella and Dire Dawa regions had same level of refusal to wife beating as equal level as that of women living in Tigray region.

**Table 2:** Binary logistic regression results of refusing wife beating among women of reproductive age

Variables	Classification	Rural		Urban	
		Number of cases	Adjusted odd ratios	Number of cases	Adjusted odd ratios
		I	II	III	IV
Region	Tigray	1339	ref	386	ref
	Afar	1027	0.66** (.13)	251	1.33 (.18)
	Amhara	1809	0.71***(.09)	259	0.66* (.17)
	Oromia	1766	1.09 (.10)	354	1.73** (.16)
	Somalia	581	0.40***(.16)	307	0.60** (.19)
	Ben-Gumuz	1083	1.41** (.10)	169	1.55* (.20)
	SNNP	1784	0.64***(.11)	238	1.06 (.19)
	Gambella	898	0.84 (.12)	226	0.83 (.19)
	Harari	442	0.68* (.15)	649	1.13 (.14)
	Addis Ababa	–	–	1724	3.05***(.13)
	Dire Dawa	368	1.00 (.15)	722	1.84***(.14)
Level of education	No Education	7070	ref	1133	ref
	Primary	3681	1.49***(.06)	2139	1.61***(.09)
	Secondary	243	2.27***(.14)	1140	2.70***(.11)
	Higher	103	7.54***(.23)	873	5.12***(.13)
Age	15–24	4376	0.73***(.08)	2427	0.83***(.09)
	25–34	3513	ref	1729	ref
	35–49	3208	1.12***(.07)	1129	1.01 (.10)
Access to media	Not at all	5269	ref	451	ref
	Sometimes	3791	0.97 (.05)	1335	1.29 (.13)
	Frequently	2037	1.06 (.07)	3499	1.49** (.13)
Workstatus	Not working	5519	ref	2386	ref
	Family business	2382	0.95 (.05)	465	1.17 (.11)
	Employed	3196	0.96 (.04)	2434	0.90 (.07)
Religion	Orthodox	3901	ref	3053	ref
	Protestant	2317	0.79** (.09)	610	0.94 (.11)
	Muslim	4525	1.12 (.07)	1572	0.68***(.08)
	Others	354	0.67** (.16)	50	0.80 (.33)
Number of living children	None	3134	ref	2590	ref
	1–2	2619	0.99 (.09)	1604	1.30* (.11)
	3–4	2447	0.73** (.11)	685	1.27 (.14)
	5 and above	2897	0.78* (.11)	406	1.21 (.17)
Household wealth Status	Low	4565	ref	1149	ref
	Medium	3355	1.02 (.06)	1194	1.17 (.09)
	High	3177	1.07 (.06)	2942	1.47***(.09)
Marital status	Never married	2227	1.54***(.09)	2153	1.17 (.11)
	Currently in union	7710	ref	2405	ref
	Ever married	1160	1.27** (.08)	727	1.01 (.10)
Intercept			0.35***(.12)		0.29*** (.20)
Total		11,097		5285	

*p < 0.05; **p < 0.01; ***p < 0.001

In urban areas, the likelihood of refusing wife beating was significantly higher in Addis Ababa, Dire Dawa, Oromia and Benishangul Gumuz regions. Numerically speaking, the attitude towards refusing wife beating is two times higher in Addis Ababa (*P* < 0.001), 84% higher in urban Dire Dawa (*P* < 0.001), 73% higher in urban Oromia (*P* < 0.01) and 55% higher in urban Benishangul Gumuz (*P* < 0.05) when compared to women living in urban Tigray that was taken as a reference (Table 2, Panel II). Nevertheless, women living in urban areas of Somali and Amhara regions have shown a significantly less likelihood of refusing wife beating. That is, their refusal attitudes had decreased by 40 and 34%, respectively (Table 2, Panel IV). What is more, women living in other urban areas of Afar, SNNP, Gambella and Harari regions had adopted similar attitudes towards wife beating as was observed in those women living in urban Tigray region.

In the vitality education was found to occur in direct relationship with attitudes towards refusing wife beating both in rural and urban Ethiopia. Explicitly, women with higher levels of education were 7.5 and 5.12 times more likely to refuse wife beating than those with no education in rural and urban areas, respectively (p < 0.001) (Table 2, Panels II and IV). Compared to women with no education, the likelihood of refusing wife beating had increased by 127 and 170%, respectively, among rural and urban women with secondary level education. The likelihood of refusing wife beating was also higher by 49 and 61% among women who attained primary level education in rural and urban areas, respectively (*P* < 0.001) (Table 2, Panels II and IV).

Younger women (15–24 years) living in rural and urban areas were 27 and 17% less likely to refuse wife beating, respectively, (*P* < 0.001) compared to women in peak reproductive ages (25–34 years). Only 12% rural women of older age (35–49 years) were in a position more likely to refuse wife beating (*P* < 0.001). Similarly, only urban women with frequent access to media and who were living in better off households were 49 and 47% more likely to refuse wife beating (*P* < 0.01) compared to women having no access to media and those living in poor households, respectively (Table 2 Panel IV). Rural women who were following Protestant and other religions such as Catholic and traditional beliefs were found 21 and 33% less likely to refuse wife beating, respectively; whereas, only 32% of Muslim women living in urban areas were less likely to refuse wife beating (*P* < 0.01).

Increasing the number of living children decreases rural women’s likelihood of refusing wife beating; whilst having no marriage commitment (i.e. never getting married to and/or dissolving a previous marriage) tended to increase the chances of women’s refusal to wife beating in rural areas (Table 2 Panel II). Contrasting this finding, having one or two living children tends to maximise refusal attitudes towards wife beating in urban areas (Table 2 Panel IV).

## Discussion

Wife beating is one of the most common and wide spread forms of GBV in the world [24]. It has affected the health and wellbeing of women, and it has resulted in countless severe socio-economic and psychological consequences. It deteriorates the social interaction between couples and among family members. It has also diminished the self-worth of women [9]. Wife beating perpetuates masculinity and male chauvinism, and it has contributed to the violation of human rights including the liberty of women to enjoy conjugal life [19]. This study revealed that region of dwelling, a proxy variable for differences in socio-cultural setting and commitment to ensure gender equity and equality, level of education, access to media, the number of living children, religion and marital status can explain attitudes towards wife beating in rural and urban Ethiopia. The following section is an attempt to put these findings into frames of references.

### Variation in attitudes towards refusal of wife beating across regions is the reflection of the diverse socio-cultural settings in the country

In Ethiopia wife-beating is a socio-culturally accepted norm that is commonly taken as the husbands’ right to “correct” erring wives through corporal punishment. Wife beating in Ethiopia is, basically, the outcome of the historically descended patriarchal system which is based on the prevalence of divisive gender roles and social norms that allows men to “discipline” their wives by using force. Wife-beating is also accepted by most Ethiopian women who live in rural areas as it falls within the culturally acceptable limits of endorsement by members of the family, the community, law enforcing agencies and health service providers, to mention some [57, 58]. In traditional societies like Ethiopia where customary rules and regulations have still the upper hands in managing social relationships and interactions, the practice is both acceptable and “legitimate”.

A couple of researches conducted in Egypt, Brazil, Ghana, Kenya and Chile have also reveal that wife-beating is an acceptable socio-cultural norm, and men in each of these countries do ‘discipline’ their wives under certain circumstances [59]. Refusing wife beating is, however, a manifestation of the social, cultural and behavioural transformation of a given society in its evolution towards a more gender egalitarian society. The variations in attitude towards refusing wife beating among women of reproductive age across regions in Ethiopia are, thus, believed to be the reflection of the socio-cultural differences in women’s status and decision making empowerment within their jurisdiction. As shown in the results of the multivariate analyses, women living in urban and rural areas of Somali and Amhara regions are not resistant to wife beating, for Amhara and Somali cultures are suppressive of women, and they provide little dignity [60, 61]. Unlike this, the Oromo culture recognizes women’s right, and it has ample mechanisms of protecting their rights and interests [62]. For instance, the *sinqe* institution preserves Oromo women’s right, and it protects their right against violations [63]. Besides, the Oromo marriage system that involves exchanges of resources also serves as a vital mechanism to protect women from being humiliated by their husbands [64].

### Education increases resistance against wife beating

Education, being a mechanism of acquiring knowledge, developing common understanding and enhancing decision making autonomy, it appeared to have a direct relationship with resistances against wife-beating. Research findings reveal that better educated women have not accepted the socio-cultural settings that perpetuate traditionalism and submissiveness rather guided by logic and common understanding based on modern and rational thinking. The inverse relationship between educational attainment and dependency syndrome of wives on their husbands to earn their living has also accelerated a better educated women’s resistance to wife beating. It is clearly identified that education is not only one of the best indicators of women’s status in the society [32], but also the other effective and powerful means of ending violence against women. The direct relationship between education and resistance against wife beating is also a reflection of education’s power to diminish socio-cultural factors [29] that perpetuate masculinity in traditional societies like Ethiopia.

### Refusal of wife beating is a function of, among other things, women’s age, number of dependent children and marital commitment in Ethiopia

As evidenced in the results of the multivariate analysis, younger Ethiopian women living in rural and urban areas have less likelihood of refusing wife beating, for they have not yet been fully integrated to accept the conjugal relationship. Being unfamiliar to the viro-local residential arrangement and stranger to the community, newly wed and young Ethiopian wives seem more likely to accept wife beating more than the others. Their courage and power to resist any sort of violence against women are often diminished by their unfamiliarity with the environment, the cultural setting and life style arrangements to which most of them are new as the dominant exogamous marriage system makes women to move to their husbands’ place [65]. The tendency to refuse wife beating will, however, have increased as they get older and establish stronger social and economic networks with their neighbourhoods and members of the community in which they live. This reveals that gaining self-confidence and protecting own interest is the function of getting familiarity with and establishing social-networking in the community. Contrary to this, refusal of wife beating or fighting against physical violence is less observed among mothers having many children, who might have been facing the economic burden to raise them as single parent who has to discharge all parental responsibilities. The tendency to jointly raise children has been discovered to expose mothers of the many children to endure recurring physical and domestic violence more often than others who have fewer or no child. Similarly, women who had been living in marital union at the time of the survey were found to be more tolerant to intimate partners’ violence; whilst those who had not yet married or dissolved their marriage were more likely to refuse the abuse. This is believed to be the effect of the socio-cultural influences which have been supported by traditional norms and values that have allowed Ethiopian males to ‘discipline’ their wives and correct them to their wants.

### Refusal of wife beating is partly deteriorated by religious faiths and rural way of life

Although no evidence is documented for the persistence of gender inequality in the Holy Bible and Holy Quran [66], followers of different religious doctrines were found to respond differently to the attitudes towards wife beating refusal. In the endeavour, it has become very interesting to observe the followers of the Protestant religion in rural areas, and the Muslims in urban Ethiopia with the less likelihood of refusing wife beating. This could be due to the re-construction of identities of Islamic religion in urban Ethiopia with strong commitment of women’s faithfulness to their husbands as a result of the on-going Islamic reform movement in the country [67]. Recent penetration of Protestant religion in to rural Ethiopia has also appeared to increase women’s loyalty to their husbands in meeting their demands without making inquiries about their rights [68]. This situation has appeared to consolidate traditional norms and values and religious preaching that have suppressed the position of women and put them under the subordinate orders of the socio-economic rank. The tendency to accept wife beating had also widely appeared to persist in rural areas than in urban localities where the traditional norms and values pervasively prevail. Hence, it is possible to argue that refusal of wife beating in the context of Ethiopia is more due to the influences of modernization, because accepting wife beating is found to mainly be a function of traditional norms and values perpetuating male supremacy.

## Conclusion

Most of the studies in developing countries on wife-beating have focused mainly on the actual prevalence of physical violence and its determinants rather than the underlying attitudes towards wife beating. Unlikely, this study took the advantage of women’s responses about their attitudes towards refusing wife beating for any of the reasons mentioned in the 2011 Ethiopian Demographic and Health Survey. Having knowing the extent of the reasons for refusing wife-beating, the researchers believed that remedial mechanisms of changing the social and cultural practices of Ethiopian societies to abolish gender based violence, in general, and wife beating, in particular, could be devised. Moreover, understanding the underlying attitudes towards wife-beating refusal is supposed to assist in designing effective and efficient intervention programmes that could address this perilous subject. Some of the intervention mechanisms that should be included could be promoting gender equity and equality and IEC campaigns to enhance men’s involvement in the initiatives to abolish GBV including wife beating. Increasing women’s access to educational and employment opportunities are also believed to bring the most significant impacts. These not only can enhance the status of women that shall narrow the gender gap between couples who share household responsibilities and resources but also resist against the violations of their rights. In addition, there is a strong need to revise and strengthen national laws and policies that protect human rights, in general, and women’s right, in particular, with a major emphasis on eradicating gender based violence. Giving due consideration to traditional and customarily rules and regulations that promote gender equity and equality, and strengthening the support of religious institutions in fostering equal opportunities for all irrespective of sex could also help to achieve the targets of ending violence against women.

### Limitations of the study

A detailed understanding of attitudes towards wife beating requires utilisation of a broad set of indicators that can capture multiple dimensions of women’s views about a woman’s hitting and beating by a husband. In this regard, indicators of perception towards wife beating used in this study may not catch the underlying causes and multiple dimensions of wife beating. In addition, the dependent variable was constructed based on hypothetical questions concerned with wife hitting and beating over certain particular causes, for capturing as many causes of conflict as possible would be unattainable. Likewise, the findings are believed to show factors associated to attitudes towards wife beating and its correlation with a set of socio-demographic, economic and cultural variables. This may enable interested institutions to design and implement proper intervention strategies to tackle and reduce the socially destructive power of masculinity. The study can also be used as an input for further research on the subject matter of interest in vitality.
